# Intra-cage dynamics of molecular hydrogen confined in cages of two different dimensions of clathrate hydrates

**DOI:** 10.1038/srep27417

**Published:** 2016-06-07

**Authors:** Margarita Russina, Ewout Kemner, Ferenc Mezei

**Affiliations:** 1 Helmholtz Zentrum Berlin, Hahn Meitner Platz 1, 14109 Berlin, Germany; 2 European Spallation Source ESS AB, P. O. BOX 176, 22100 Lund, Sweden; 3 Wigner Research Center for Physics, P. O. BOX 49, 1525 Budapest, Hungary

## Abstract

In porous materials the molecular confinement is often realized by means of weak Van der Waals interactions between the molecule and the pore surface. The understanding of the mechanism of such interactions is important for a number of applications. In order to establish the role of the confinement size we have studied the microscopic dynamics of molecular hydrogen stored in the nanocages of clathrate hydrates of two different dimensions. We have found that by varying the size of the pore the diffusive mobility of confined hydrogen can be modified in both directions, i.e. reduced or enhanced compared to that in the bulk solid at the same temperatures. In the small cages with a mean crystallographic radius of 3.95 Å the confinement reduces diffusive mobility by orders of magnitude. In contrast, in large cages with a mean radius of 4.75 Å hydrogen molecules displays diffusive jump motion between different equilibrium sites inside the cages, visible at temperatures where bulk H_2_ is solid. The localization of H_2_ molecules observed in small cages can promote improved functional properties valuable for hydrogen storage applications.

Understanding the mobility and dynamic activity of molecules and ions in confinement is highly significant both from academic point of view and for the broad range of applications based on the confinement of ions and molecules, such as hydrogen storage or development of ionic conducting materials and supercapacitors. In porous materials the molecular confinement is often realized by means of weak Van der Waals interactions between the stored molecule and the pore surface. Thus the interaction between guest and matrix can be expected to be sensitively influenced by structure of the confining host, in particular the dimension of the confinement cages. Recent studies reported the impact of the pore size on the storage capacities for hydrogen^1^ and ions^2^ confined in porous carbon with higher storage capacities observed for the small pores. However, little is known about the microscopic mechanism of such effects.

Clathrate hydrates are one group of nanoporous materials with a high potential use for hydrogen storage. The advantage of such materials is that they can keep large quantities of gas at elevated temperatures and at ambient pressure, while being environmentally benign materials. Discovered first in 1999^3^, hydrogen clathrate hydrates are created from ice under high hydrogen pressure as a result of interactions between hydrogen and water molecules, which push the ice to change its microscopic structure and create cages of different dimensions to accommodate the H_2_ guest molecules^4^. The hydrogen filled clathrates form structure sII (cf. Fig. 1), consisting of two types of cages^4^, i.e. “small” dodecahedron 5^12^ cages with a mean crystallographic radius of 3.95 Å and “large” hexakaidecahedron 6^4^5^12^ with a mean radius of 4.75 Å. The accessible pore volume is however smaller considering the van der Waals radii of the oxygen atoms and is of about 1.9–2.7 Å average radius in the small cage and about 3.3–3.5 Å in the large one. While the small cage, as a rule, accommodates one H_2_ molecule, the large cage can accommodate 2–4 H_2_ molecules depending on the conditions applied^5^,^6^ with the maximum storage capacity reaching 3.77 wt%. In pure clathrates, where H_2_ occupies both cages, the clathrate is synthetized and loaded by gas at 2000 bars at 250 K^6^. Under ambient pressure, the process of deintercalation of H_2_ molecules starts with increasing temperature at 70 K with the consequent collapse of the clathrate structure around 170 K^5^. In so-called binary clathrates large cages are occupied by large molecules such as tetrahydrofuran^7^ and the filling of the remaining small cages by H_2_ gas is realized already at a pressure of 60 bar of H_2_ at 260 K. The starting temperature of H_2_ deintercalation in such clathrates increases to 255 K under ambient pressure^8^. The observed differences in H_2_ sorption pressure and temperature of the gas deintercalation of these two types of clathrates raise the question whether they can be related to the differences in interaction between hydrogen and host framework inside the different size cages? The direct observation of the motion on the microscopic scale of the hydrogen molecules can be an essential part of the answer.

In general, a H_2_ molecule in a solid or in a confinement can take part in various types of motions: rotations around its center of mass, vibrations around an equilibrium position occupied for much longer time than the period of the vibrations and jumps between equilibrium positions. The last type of motion is generically called self-diffusion^9^ and has been both experimentally and theoretically extensively studied in great detail in many systems^9^,^10^. Note that diffusion inside closed confinement domains does not imply macroscopic material transport. On the other hand, if the diffusive motion inside confinement cages is slow, this can slow down macroscopic diffusion implying transitions from one cage to another through their common surface^9^. Thus, the macroscopic cage-to-cage migration of H_2_ molecules depends on several aspects of the behavior and interactions of the H_2_ molecule, including the mobility inside the confinement cages and the activation energy needed for molecules to pass from one cage to another. Previous studies determined such activation energy values^11^,^12^ to pass through the pentagon and hexagon windows separating the cages as 26 kcal/mol and 6 kcal/mol respectively, but were unable to distinguish experimentally between the motion of the hydrogen inside different cages^8^,^12^. The experimental estimates of the long range self-diffusion constant for hydrogen contained in small cages of binary clathrates range^13^,^14^ from 10^−8^ cm^2^/s to 10^−11^ cm^2^/s. Later, it was claimed that hydrogen does not diffuse there at all^15^. The cage-to-cage diffusion of confined H_2_ and stability of entire clathrates hydrates have been also studied as a function of cage occupancy with help of molecular dynamic simulations^16^,^17^. It was found that the increase of the number of occupants leads to the higher mobility. In the small cage the double occupancy results in the distortion of the unit cell and is thermodynamically unstable leading to the expulsion of one molecule^16^ out of the cage. In the large cage higher number of confined molecules is claimed to reduce the energy barrier of the hexagonal window due to the guest-host interactions^17^.

Rotational motion of confined hydrogen has strong quantum features. Quantized rotation transitions *E*_*J*_ of molecular hydrogen with respect to the ground state are given by *E*_*J*_ = *BJ*(*J* + 1)], where B = 7.35 meV is the quantum rotational constant and J is a quantum number J = 0, 1…. 17. The confinement leads very often to the lifting of degeneracy of states and, indeed, such splitting of rotational transitions has been reported for H_2_ in both cages in hydrogen clathrates^18^,^19^,^20^. Vibrational motion of confined hydrogen can be both localized and induced by host lattice phonons depending on the coupling to the clathrates hydrates framework. The coupling of the H_2_ and clathrates hydrates is expected to be weak, however, due to the small size of the molecules^20^,^21^,^22^. There is a rather generic notion of “rattling” motion of guest molecules inside the cage, often defined as large-amplitude anharmonic vibrations of the guest molecules^22^,^23^,^24^. In hydrogen filled clathrates the existence of quantum rattling at frequencies centered around 10 meV (80 cm^−1^) has been reported for the hydrogen enclosed in the small cage^18^,^19^,^20^,^21^,^25^, while the existence of such modes in large cage was not confirmed experimentally yet.

The goal of the presented work is the study the role of the confinement dimensions in the intra-cage dynamics of molecular hydrogen enclosed in nanocages of two different dimensions in clathrate hydrates. Besides high technological interest clathrate hydrates are particularly suitable model systems to study the role of confinement since the interactions between the framework of the clathrates and H_2_ are of the same nature in both cages. For experimental characterization we used neutron quasielastic scattering due to its remarkable capability to probe directly and specifically the individual motion of H_2_ molecules at nanoscale. To distinguish between the contributions of hydrogen in two different cages we used a combination of data from binary tetrahydrofuran and fully hydrogenated clathrates. The signal of hydrogen confined in small cages was established using the difference between the empty and H_2_ gas loaded spectra of binary tetrahydrofuran clathrate, where H_2_ only occupies small cages. Subtracting the small cage H_2_ signal from the spectrum of pure hydrogen clathrates with H_2_ in both small and large cages, we can deduce the signal of H_2_ molecules in the large cages. This method of course is a first approximation, and assumes that the occupation of neighboring cages has little impact on the confined particle motion. In view of the sII structure one can expect that this is a good approximation^5^,^20^,^22^, and indeed it provided us with a self-consistent ensemble of results. The average occupation of hydrogen in our study was found to be one molecule in the small cage and two in the large ones.

It has to be mentioned that in general the spectral intensities in molecular hydrogen depend on the composition in terms of ortho and para spin isomer states, which can be altered by the relaxation between these states. In the experiment we verified that the concentration of ortho-hydrogen stayed constant by immediately cooling down the samples after H_2_ loading to 10 K and measuring the scattering intensities, and periodically repeating this process. We found the signal unchanged over the duration of the experiment, confirming our earlier systematic observation of slowdown of ortho to para hydrogen conversion due to confinement. We can thus assume that the stored H_2_ remained in the normal 3:1 ortho-para concentration ratio, which is the equilibrium value at the loading temperature of 250 K. Since, in addition, the neutron scattering cross section of ortho-hydrogen is nearly two orders of magnitude higher in the (*Q*, *ω*) domain of our study than for para-H_2_, in the data analysis we could assume that within error all the signal comes from scattering on ortho-hydrogen.

## Results and Discussion

The feature measured in neutron spectroscopy is the dynamic structure factor *S*(***Q***, *ω*) that is the Fourier transform of Van Hove space-time correlation function, weighted by the scattering strength of the various atomic nuclei (here 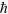
*ω* stands for the neutron energy transfer and ***Q*** = ***k***_*f*_ – ***k***_*i*_ for the neutron momentum transfer)^26^. Due to the very large incoherent scattering cross section of hydrogen the dynamic structure factor is dominated by the signal related to the self-correlation function of molecular hydrogen.

For the analysis contributions of various types of motion of H_2_ molecules have to be taken into account, which can be written as a convolution^9^:


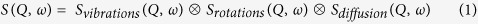


In the temperature and energy range studied, vibrations contribute to the spectra through a Debye-Waller factor *f*_*DW*_, which in the case of isotropic mean square displacement of the center of mass <*u*^2^> is equal to 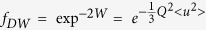. In the temperature and momentum range of our experiment this factor was consistently found to remain close to 1. The inelastic rotation contribution consists of the spectrum of transitions between quantized rotational states *J* = 0, 1, …, the first of which above the *J* = 0 ground state occurring at *E* = 14.7 meV, i.e. outside the energy range studied in this experiment. The remaining elastic scattering component in *S*_*rotations*_(*Q*, *ω*) for the ground state of molecular hydrogen can be described by^27^,^28^


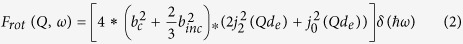


where *j*_*i*_(*Qd*_*e*_) indicates the spherical Bessel function of corresponding order, *d*_*e*_ is the equilibrium H-H distance of 0.74 Å and 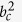
and 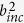 are the coherent and incoherent scattering cross sections of hydrogen. In the data analysis *S*_*diffusion*_(*Q*, *ω*) was determined by normalizing the experimental data to the rotational contribution by using eqs (1) and (2), assuming equilibrium H-H distance. The dimensionless quasielastic neutron scattering structure factor for diffusive motion we are thus concerned with is generally described as^9^:


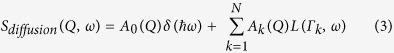


*A*_*0*_*(Q)* stands for the so-called elastic incoherent structure factor (EISF) and reflects geometrical parameters of the corresponding diffusive motion, *A*_*k*_(*Q*) are amplitudes of the corresponding quasielastic contributions of different diffusional modes and 
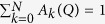
. *L*(*Γ*, *ω*) indicates a Lorentzian 
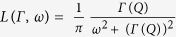
, with a width *Γ.*

Neutron scattering spectra of the bulk and confined hydrogen measured at the time-of-flight spectrometer NEAT^29^ are presented in Fig. 2 and show clearly that by varying the confinement dimension we can decrease or even increase the hydrogen mobility compared to that in bulk hydrogen at the same temperature. The melting and boiling temperatures of bulk hydrogen at ambient pressure are 13.99 K and 20.27 K, respectively^30^. In the frozen state the spectra of the bulk hydrogen at T = 10 K show the elastic line only, centered around 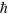
ω = 0 meV (Fig. 2a). With melting of hydrogen the elastic line is transformed into a broad Lorentzian due to more diffusive motion of the molecules. At T > 20.27 K hydrogen is a gas, which leads to faster dynamics and a strong decrease of the signal within our energy/time window.

H_2_ confined into small cages exhibits up to 50 K no signal in addition to the intense elastic line (Fig. 2b). This indicates strongly restricted dynamic activity of H_2_ in small cages, where H_2_ is practically “frozen” on the timescale of this study. Neither does the Q-dependence of elastic intensities of hydrogen in small cages show any contribution of diffusive motion and can be described by a mean square displacement of the center of mass due to rotational and vibrational contributions. The observed values of the mean square displacement range from 0.04 ± 0.07 Å at 10 K and 0.1 ± 0.07 Å at 50 K, which confirms strong localization of hydrogen in small cage. At higher temperatures the hydrogen molecules become more mobile and start to explore a small volume in the center of the cage with radii ranging from 0.5 Å at 90 K to 0.9 Å at 200 K^31^.

**Figure 1:**
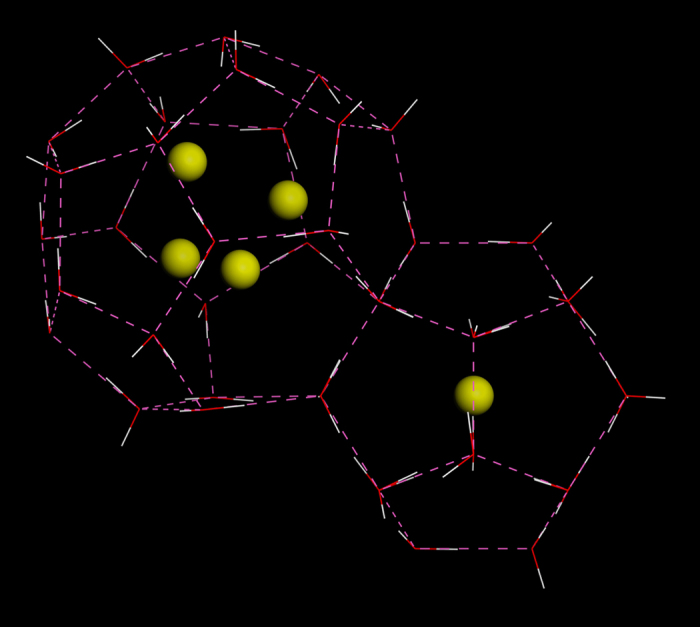
Structure of hydrogen clathrates. Hydrogen molecules are indicated by yellow spheres, framework water molecules are shown by red and white lines. Magenta dashed lines indicate hydrogen bridges. The structure of clathrate type sII is cubic with a = 17.047 Å and is formed by 8 large cages of hexakaidecahedron (6^4^5^12^) and 16 small cages of dodecahedron (5^12^) shapes with mean crystallographic radii of 4.73 Å and 3.95 Å, respectively. In the case of maximum H_2_ occupancy, the clathrate can be denoted as 48H_2_ × 136H_2_O with H_2_ storage capacity of up to 3.77 wt%. (In our samples we have replaced H_2_O by D_2_O).

**Figure 2:**
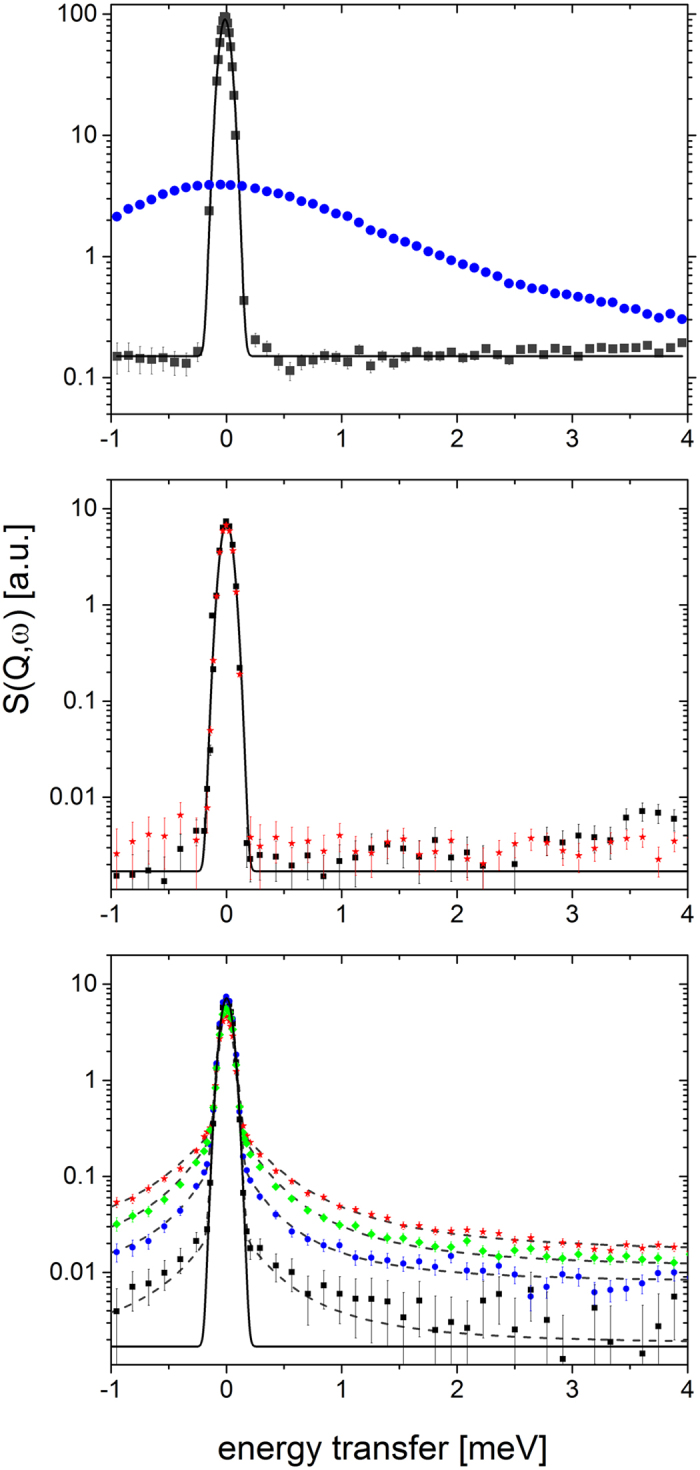
Dynamic structure factors of molecular hydrogen. (**a)** In bulk at ambient pressure, (**b)** Confined in small cages, (**c)** Confined in large cages. The solid line represents the instrumental resolution, which was measured independently with a standard elastic scatterer. Points show experimental data at *Q* = 1 Å^−1^ at different temperatures: ◼ T = 10 K, 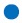 T = 20 K, 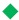 T = 30 K and 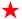 T = 50 K. Dashed lines show fits by the model in Eqn. (4). Pronounced quasielastic signal of the hydrogen confined in the large cage can be observed even at T = 10 K indicating higher mobility of confined hydrogen compared to the bulk solid at the same temperature. The energy width of QENS intensity is about constant in the experimentally covered *Q* range revealing spatially confined diffusion.

**Figure 3:**
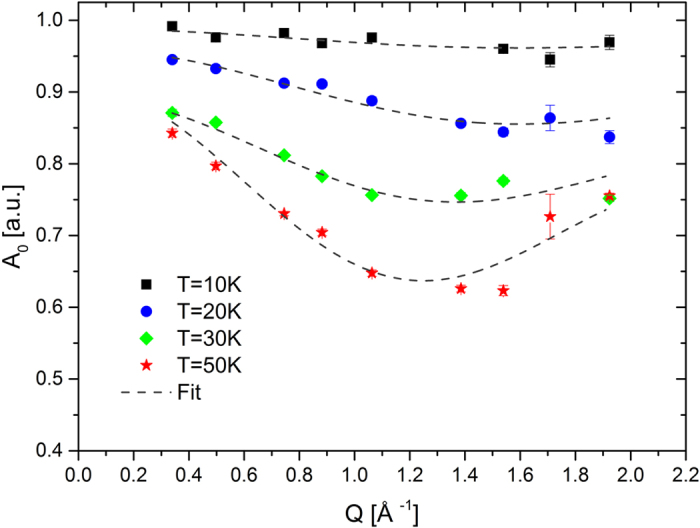
Elastic incoherent structure factor (EISF) for confined H_2_ in large cage. The momentum transfer *Q* dependence of elastic fraction *A*_*0*_ of the observed dynamic structure factor of molecular hydrogen confined in a large clathrate cage. Experimental data are represented by points, dashed lines show fits at different temperatures by the model of jump diffusion within the tetrahedral cluster of equilibrium positions and a mobile fraction increasing with temperature according to Eqn. (6), details are described in the text. A minimum around 1.3 Å^−1^ corresponds to jumps with length of about 3.45 Å between the equilibrium sites at the corners of a tetrahedron. With increasing temperature a small fraction of particles reversibly escapes the clathrate structure and cause the downwards shift towards *Q* = 0 Å^−1^ for T = 30 and 50 K.

The increase of the confinement size from 3.95 Å to 4.75 Å of the average crystallographic radius leads to the strong increase of the hydrogen mobility already at 10 K in the large cage compared to both the bulk solid and hydrogen confined in the small cage at the same temperature. As a result, in addition to the elastic line at 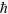
ω = 0 we observe a strong quasielastic signal (QENS) at low frequencies already at T = 10 K (Fig. 2c). We have applied several models including the diffusion on the surface of and within a volume of a sphere, jumps between two and more positions in a cage^9^ for the analysis of the data. Our results revealed that the observed signal best corresponds to jumps between different equilibrium sites located at the corners of a tetrahedron inside the large cage (Fig. 1): The hydrogen molecule rests on a site for the residence time *τ*_*s*_ and jumps toward another site placed at distance *l* during a time interval much shorter than the residence time^32^. Considering occupation of large cages by the experimentally observed average number of two molecules, the quasielastic signal for this motion can be then described^32^ by a combination of two Lorenztians with widths proportional to 1/*τ*_*s*_:


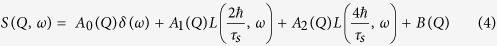


where *B*(*Q*) is the constant (ω independent) baseline. The geometrical arrangement of the equilibrium sites determines the specific form of the momentum transfer *Q* dependence of the intensity of the elastic line (EISF) *A*_*0*_*(Q)* (Fig. 3), which in our tetrahedral case can be described as


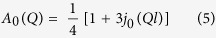


while 
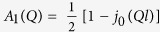
 and 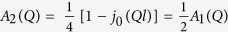. Here j_0_ is the spherical Bessel function, displaying a minimum at *Ql* = 4.5. The full quantitative analysis of the data in Fig. 3 also reveals evidence for the presence of a *Q* independent elastic signal, i.e. a fraction *K*_*imm*_ of the H_2_ molecules that do not participate in the observed diffusive motion. Thus in our model in Eqn. (4) the functions *A* are to be replaced by


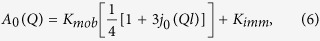



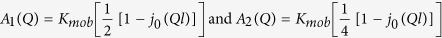


Using the set of five variable parameters at each temperature, i.e. the fractions of mobile and immobile particles *K*_*mob*_ and *K*_*imm*_, respectively, the jump length *l*, the residence time *τ*_*s*_ and the spectral baseline *B*(*Q*), we were able to well fit all spectra in the (*Q*, *ω*) range covered experimentally, as represented by the dashed lines in Figs 2c and 3. In contrast, the application of other models did not lead to satisfying results. Particularly, the model for the jumps between two sites (dumb-bell) reproduced well the Q dependence of the EISF (Fig. 3), but was not able to provide consistent results for the temperature dependence of the spectral lineshape in the range studied (Fig. 2c). The determined parameters *τ*_*s*_ and *l* for all temperatures are summarized in the Table 1.

It is significant, that the jump length deduced from our data in Fig. 3 are in remarkable agreement within error (given in Table 1 as standard deviation) with the distances of 2.93 Å between the 4 tetrahedrally arranged equilibrium sites for H_2_ (actually D_2_) molecules found by the crystallographic study^5^. This study has also found that the H_2_ molecules in the large cages can be both in localized or delocalized states and the fractions in each state is a function of temperature and pressure in equilibrium. This lends strong principal support for our observation of mobile and immobile fractions of H_2_ atoms, even if the pressure, temperature and loading parameter domain explored there does not overlap with ours in this work. The distance between the equilibrium sites (i.e. the jump length *l*) is on the other hand significantly shorter than the H_2_–H_2_ distance of 3.776 Å in solid hcp hydrogen crystal at ambient pressure^33^, indicating that this confinement leads to more compressed but also more mobile state than solid hydrogen.

The obtained values of the residence time *τ*_*s*_ fall in the range of those reported previously for hydrogen adsorbed on the carbon nanohorns^27^, however show much weaker temperature dependence. The observed difference in mobility between small and large cages can be understood as caused by the modulation of cage potentials as a function of the cage dimension. The localization of hydrogen in small cages indicates the existence of molecular traps of potential minima in the center of the cage, matching the molecule size. The trapping can explain the reduction of the sorption pressure which is required for the loading of hydrogen molecules inside the small cage. In the same time it helps to contain the hydrogen inside the cage and enhances in this way gas release temperature in binary clathrates. The increase of the cage dimension leads to a flatter potential that, in contrast, promotes intra-cage mobility. Indeed, the weak temperature dependence of residence time *τ*_*s*_ indicates low values of activation energy required for molecules to move between four equilibrium positions. Furthermore, our findings are supported by previous theoretical calculations which revealed deep minima in the potential energy surfaces of small cages with a width of about 1.5–2 Å^25^,^34^ and a flattening of potential in the large cages^34^ with observed off-center minima at about the distance of 3 Å from each other.

The glass type mobile–immobile dynamic heterogeneity^35^ observed in a well-ordered crystalline matter and present at all temperatures is a clear signature of substantial randomness in the flatter potential landscape in the large cages. This randomness can be caused by spread in hydrogen bond lengths and angles in the host structure, as reported recently^36^. In addition, the random occupation of the four H_2_ equilibrium sites in the large cages filled on average by two hydrogen molecules can lead as well to potential fluctuations and higher disorder. Random inhomogeneities in the potential landscape are widespread in porous systems; therefore the existence of dynamic heterogeneity is expected to be a common feature, which has to be considered when conceiving new materials. The representation of such dynamic inhomogeneity by two extremes, a mobile fraction with a given residence time and an immobile fraction with at least an order of magnitude longer residence time (which would remain hidden within experimental resolution) can just be the simplest quantitative interpretation. A dynamic heterogeneity in the form of broad distribution of residence times caused by the random variations in the structure would lead to very similar spectra, in particular in view of the temperature dependent flat background B(Q), which could contain contributions from long spectral tails resulting of a broad distributions of Lorentzian line widths in the spectrum.

In summary, we found direct evidence of large difference in the microscopic dynamic behavior of molecular hydrogen confined inside cages of different dimensions in the nanoporous clathrate hydrates. In the small cages of clathrate hydrates with average crystallographic radius of 3.95 Å we observe a structural arrest of confined hydrogen that can play a substantial role in determining the functional properties such as reducing of the sorption pressure of hydrogen and enhancing of the gas release temperature. The moderate increase of the crystallographic confinement radius to 4.75 Å for the large cages leads, in contrast, to a formation of novel type of hydrogen state with a shorter H_2_–H_2_ distance but at the same time substantially higher mobility at T = 10 K then the bulk hcp hydrogen at the same (ambient) pressure and the same temperature. Crystallographic evidence shows^5^ that H_2_ molecules in the large cages start to delocalize within the cages with increasing temperature at 70 K and to escape at ambient pressure from the high pressure loaded clathrate hydrate at 100 K. Our observation of significant diffusive mobility in the large cages at 10–50 K temperatures suggest that the macroscopic diffusion should rather be governed by the activation of the molecular transitions between large cages at higher temperatures. The H_2_ molecules confined in the small cages, on the other hand, are trapped in the same temperature range at the center of the cages and might not be available for long range diffusion, independently of the activation of inter-cage jumps. This is an example for the confined mobility in the cages to influence the diffusion between the cages, which is responsible for the macroscopic material transport. Furthermore, in the large cage we observe strong glass-like dynamic heterogeneity which can be explained by a significant disorder of the potential landscape in crystalline clathrate network. Our direct observation of motion of H_2_ molecules inside the cages of clathrate hydrates give direct evidence and a space-time characterization of the dynamic activity that constitutes or contributes to what phenomenologically is often referred to as “rattling”. Our study reveals large quantitative and qualitative impact of the dimension and finer details of the confinement structure on functionally relevant dynamic behavior of the stored molecules. Similar effects can play a role in recently observed strong decrease of the ionic current and the non-linear dependence between the current and applied potential for smaller pores in ionic systems confined in porous carbon^37^,^38^. Confined ions can become localized in the potential wells as the pore diameter becomes comparable to the size of the solvated ion thus higher energy penalty is needed to extract ions from the pore.

## Methods

### Sample preparation and neutron spectroscopy experiments

The clathrates samples were prepared using fine, 99.8% deuterated ice powder. The preparation was done following the procedures described before^31^,^39^. For the synthesis of the binary tetrahydrofuran clathrates we added deuterated tetrahydrofuran (99.5%) to deuterated water in stoichiometric proportion (17:1 mol). The solution has been stirred in a thermal bath at T = 275 K for 48 hours until crystallization occurs. Prepared ice samples were ground to fine powder and loaded into a precooled cylindrical cell under cold nitrogen atmosphere and pressurized by hydrogen gas for 24 hours at temperature cycled in 270–277 K range. For the synthesis of pure hydrogen clathrates we have applied the pressure of 2000 bars, while for loading of TDF clathrates we used 200 bars. Afterwards, the samples have been cooled down slowly to 20 K by keeping the H_2_ pressure at 200 bars for another day. Using the protection of cold nitrogen atmosphere at 1 bar pressure, the prepared samples have been loaded into aluminum flat cells, sealed and placed into the cold cryostat at T = 30–50K for neutron scattering investigations. The weight of the samples has been monitored before and after the experiment.

By using deuterated hydrogen containing materials in the samples with the exception of the fully protonated H_2_ gas filling we have achieved that the incoherent neutron scattering cross section was to >80% dominated by the signal from the loaded H_2_ gas. In addition, the spectra measured on the H_2_ unloaded tetrahydrofuran clathrate were used as background for correcting the data for the signal from the D_2_O matrix and tetrahydrofuran.

The neutron scattering experiments were done at the time-of-flight spectrometer NEAT at Helmholtz Zentrum Berlin^29^ using two experimental configurations. The first one with incoming neutron wavelength λ_I_ = 2 Å has been used to probe diffraction patterns, which monitored the formation of the clathrates. The second configuration with λ_I_ = 5.1 Å and instrumental elastic resolution ranging between 90 and 110 μeV has been used for investigation of the dynamics in the low energy range (−2 to 5 meV neutron energy transfer) corresponding to picosecond time scale. The spectra, collected in the temperature range from 10–50 K, were corrected and evaluated using standard data treatment routines. In addition, for the data analysis we excluded detector areas where we observed Bragg reflections from the clathrate framework.

## Additional Information

**How to cite this article**: Russina, M. *et al.* Intra-cage dynamics of molecular hydrogen confined in cages of two different dimensions of clathrate hydrates. *Sci. Rep.* **6**, 27417; doi: 10.1038/srep27417 (2016).

## Figures and Tables

**Table 1:** Parameters of diffusive motion for H_2_ confined in large cages.

Temperature [K]	Mobile fraction [%]	Jump length *l* [Å]	Residence time *τ*_*s*_ [ps]
10	4 ± 1	2.4 ± 0.6	6.0 ± 0.13
20	16 ± 1	2.4 ± 0.2	6.1 ± 0.19
30	21 ± 2.2	3.22 ± 0.2	6.3 ± 0.16
50	36.5 ± 2.2	3.6 ± 0.3	4.4 ± 0.13

Temperature dependence of the mobile fraction *K*_*mob*_/(*K*_*mob*_ + *K*_*imm*_), jump length *l*, and residence time *τ*_*s*_ (deduced from a larger body of data than shown in the figures).
